# The Relationship between Emotionally Laden Landmarks, Spatial Abilities, and Personality Traits: An Exploratory Study

**DOI:** 10.3390/brainsci10060326

**Published:** 2020-05-27

**Authors:** Francesco Ruotolo, Filomena L. Sbordone, Ineke J.M. van der Ham

**Affiliations:** 1 Laboratory of Cognitive Science and Immersive Virtual Reality, Department of Psychology, University of Campania “L. Vanvitelli”, Viale Ellittico, 81100 Caserta, Italy; filomenaleonela.sbordone@unicampania.it; 2 Department of Health, Medical and Neuropsychology, Leiden University, Leiden 2333 AK, The Netherlands; c.j.m.van.der.ham@fsw.leidenuniv.nl

**Keywords:** spatial memory, emotions, individual differences

## Abstract

Separate research lines have shown that the way we process spatial information is influenced by individual factors, such as personality traits and basic spatial abilities. Alongside, recent studies suggest that environmental landmarks can be represented differently depending on their emotional content. However, to our knowledge, no study has addressed so far the issue of whether there is a relationship between individual factors and the way we represent and use spatial information that conveys emotional content. Therefore, this exploratory study aimed to (i) investigate the relationship between personality traits and the use of spatial strategies in relation to emotional stimuli; (ii) investigate if a different pattern emerges according to a body- or object-based spatial encodings. After watching movies of routes characterized by positive, negative, or neutral landmarks, participants performed a “route continuation” (RC, i.e., left/right decision) and a “distance comparison” task (DC, i.e., what was the landmark closest to X?). Furthermore, participants performed a mental rotation task (MR), the Corsi block tapping (CBT), and the Bergen right-left discrimination tests (B-RL). Personality traits were assessed through the Ten Item Personality Inventory (TIPI). Results showed that a better performance at the RC task was associated with higher scores at CBT tasks in the positive condition and at B-RL test and agreeableness scale from TIPI in both positive and neutral conditions. Instead, the MR task positively correlated with the DC task in all conditions. In sum, individuals’ spatial abilities, personality traits, and task requests influenced the way emotionally laden landmarks were memorized.

## 1. Introduction

Very often, during the exploration of the environment, we come across elements that can have a positive (e.g., a beautiful villa or urban park) or negative valence (e.g., a degraded place, an overturned garbage can or an episode of violence). These elements can represent landmarks to which we attribute a personal dimension (cf. [1]) and can be used according to specific spatial strategies: represent a route and orient ourselves during navigation (e.g., << I remember that in front of this beautiful house we turned right >>). Interestingly, it has been shown that these landmarks may differently affect our spatial memory according to their valence (i.e., positive, negative, or neutral). For example, Balaban and colleagues [2] showed that people were more accurate in remembering directional information (i.e., did you do left or right at landmark X?) associated with negative rather than neutral landmarks. Instead, Piccardi and colleagues [3] found that participants exposed to both negative and positive rather than neutral landmarks were faster in learning the path connecting them (see also [4]). Finally, Ruotolo and colleagues [5] showed that the presence of positive, but not negative, landmarks positioned along a path improved the accuracy of the mental representations of the distances between them and the memory of their absolute position along the path. Moreover, the path was perceived as longer in the presence of negative, rather than positive and neutral landmarks.

In sum, these studies indicate that the influence of emotionally laden landmarks on route representation may vary according to their valence (i.e., positive, negative, or neutral). Specifically, they would suggest that negative landmarks affect mostly observer-based spatial information (e.g., right/left turn information, length of perceived distances), whereas the positive ones seem to facilitate object-based spatial representations (e.g., distances between landmarks and their reciprocal position). However, whatever the effects produced by this type of landmarks, the processes/mechanisms at the basis of these effects are still unknown.

One way to address this issue is to explore the role that basic spatial abilities could have in the spatial representation of landmarks with different valence (e.g., positive, negative, or neutral). For example, it has been shown that individuals with high mental rotation abilities, i.e., the ability to mentally rotate an image along a continuous trajectory in the mental space until it reaches a new orientation [6,7,8], perform better than participants with low mental rotation ability in spatial navigation [9,10], wayfinding [11], orientation, and route learning tasks [12]. More importantly, Kaltner and Jansen [13] found that mental rotation performance was enhanced after the presentation of fearful images compared to neutral images. Furthermore, a link between a low performance in visuospatial working memory measured by the Corsi task and topographical disorientation has been suggested (e.g., [14,15,16,17,18]). As for the mental rotation task, Palmiero and colleagues [19] showed that participants’ performance at the Corsi task and its walkable version [20,21] improved when emotional stimuli were used as compared to the neutral ones. Finally, another cognitive skill that could be considered is the ability to discriminate left from right (left-right discrimination: LRD; [22]). When navigating through the environment, we are constantly exposed to right/left decisions (e.g., at the church I turn right, then I go left, etc.), for which left/right identification or discrimination is essential.

In addition to cognitive skills, personality traits can also influence the way we manipulate spatial information [23,24]. For example, it has been shown that extroverted people show more exploratory behavior and are more efficient in navigational tasks [25]. Similarly, Pazzaglia and colleagues [24] found that performance on wayfinding and route-tracing was positively associated with participants’ agreeableness and conscientiousness, respectively. Instead, people scoring high on psychoticism tend to perform worse in wayfinding tasks [26]. Considering personality traits in combination with the emotional content of landmarks seems a valuable addition, as it has been suggested that individuals with different personality traits can interpret emotional events differently [27,28,29].

Overall, the above-mentioned evidence suggests that individual factors, i.e., both basic spatial abilities and personality traits, can play a role in how spatial information linked to emotionally laden landmarks is represented in memory. Therefore, the aim of this work was to explore the type of relationship that might exist between basic spatial abilities, personality traits, and representations of emotionally laden landmarks. Furthermore, since it has been suggested that positive and negative stimuli can have different effects depending on whether the spatial information is more observer-based (i.e., to the direction of his/her movement) or object-based (i.e., to the relationship or distance among landmarks), this work also aimed to observe if the role of the spatial abilities and personality traits changes according to the kind of spatial information required.

To this aim, participants were requested to perform the Corsi block tapping test (CBT; both forward and reversed version), the Bergen right-left discrimination task (B-RL), a mental rotation task (MR), and to fill out the Ten item personality inventory (TIPI) [30]. Afterward, they watched three movies of a virtual walk characterized by positive, negative, or neutral landmarks and had to memorize what they saw. After each video, participants were asked to perform two spatial judgment tasks: a route continuation task (RCT, e.g., at landmark X did you do left or right?) and a distance comparison task (DCT, e.g., what was the landmark closest to “X", Y or Z?). Although the current study used some materials and procedures previously used by Ruotolo and colleagues [5], it differed from the previous one in two fundamental aspects. First, here participants explored three different paths and not only one. Second, while in Ruotolo and colleagues [5], the relationship between landmarks was investigated with a task that could stress the use of a body-based strategy (i.e., imagine walking from one landmark to another), here the relationship between landmarks was measured with a task that stressed the use of an object-based strategy.

If basic spatial abilities and personality traits play a specific role in the way spatial information of emotionally laden landmarks is memorized, then we expect significant and higher correlations between performance in both spatial tasks (i.e., RCT and DCT) and individual factors in positive and negative conditions rather than in the neutral one. Furthermore, since the CBT measures visuospatial working memory, in a sequential context, it is possible to hypothesize a positive association between performance in this task and the route continuation task. The route continuation task should be also positively associated with the ability to discriminate right and left. Instead, the performance in the MR task should be more positively associated with performance in the distance comparison task. Finally, extroverted/agreeable personality traits should be positively associated with performance in both continuation and comparison tasks. However, the few studies conducted so far on this topic do not provide enough theoretical and/or empirical knowledge to formulate more specific hypotheses about the relationship between spatial abilities, personality traits, type of spatial task, and landmarks’ valence. Therefore, on this part, the current study is largely exploratory.

## 2. Method and Materials

### 2.1. Participants

A total of 138 participants were included in the study (79 females, mean age = 22.34, SD = 2.73). Participants were all students from the University of Leiden, with a normal or corrected to normal vision. They declared they did not suffer from any psychiatric or neurological disease. Participants were recruited through notices posted on university notice boards and by word of mouth. The signed informed consent was obtained from all participants. For their participation, participants could choose to be paid (6 euros per hour) or granted course credit. The experiment was approved by the Ethics Committee of the University of Leiden (CEP16-0127/27) and was in conformity to the principles of the Declaration of Helsinki (2013).

### 2.2. Setting and Materials

#### 2.2.1. Virtual Routes

**Figure 1:**
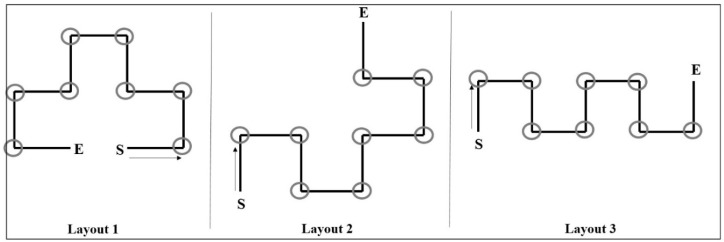
The figure depicts the three different layouts. S = starting positions; E = end of the route. The grey circles indicate where the images were put along the route. Virtual routes had the same length and temporal duration.

**Figure 2:**
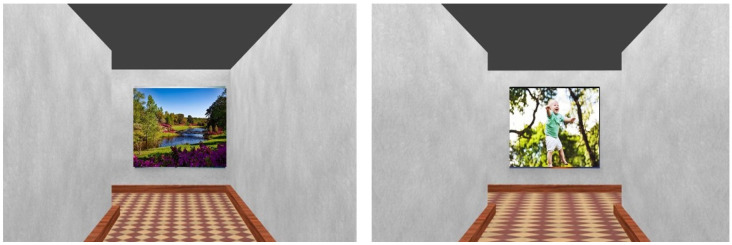
The figure depicts two examples (on the right and left) of participants’ first view perspective during the navigation of the route. Note: the images on the route’s wall are copyright-free images taken from https://www.pexels.com/it-it/. They are present in this figure just for illustration purposes.

Three different virtual routes were presented to the participants (see Figure 1). One of the virtual route (Layout 1) was already used in a previous study (i.e., [5]). The other two layouts were added to increase the external validity of the study, so as to avoid limiting the results to only one type of spatial configuration. The virtual routes were created with the Software BLENDER and were characterized by nine segments of the same length (the length was expressed in Blender units, that is, 4 units for each segment; each unit would represent 1 m). The outer structure consisted of concrete walls to generate a neutral maze. The virtual routes represented automated walks through a maze from the first-person perspective. The first-person perspective was at walking speed and 170 cm in height (see Figure 2). Virtual routes were characterized by three different layouts, but all the virtual walks lasted 103 s. Eight images/landmarks were placed along the route and centrally at the wall at the end of each corridor, with the exception of the last one where no image was attached. More importantly, in order to verify the presence of effects due to the type of layout, 26 one-way ANOVAs were carried out. Each ANOVA had as the independent variable the layout (with three levels) and as a dependent variable one of the measures adopted in this study. The results showed no significant differences among the three layouts for any of the measures considered. This indicated that the layouts had a similar level of difficulty and that the individual factors (i.e., performance at MR, CBT, B-RL tasks, and scores at TIPI) were equally distributed across the different layouts. Therefore, the difference among the layouts was no longer considered in the analyses.

#### 2.2.2. Images

All images (48 in total) were taken from the IAPS inventory (the international affective picture system; [31]). Each route/layout had three versions: one with only neutral images, one with positive images interspersed with neutral images, and one with negative images interspersed with neutral ones. The chosen images with the corresponding valence and arousal ratings and separated for each condition are presented in Appendix A (Table A1). Positive and negative images were matched for their arousal level while having different valence average values, whereas neutral images had low arousal and medium valence. Finally, 48 other images were chosen that were matched in terms of semantic content, arousal, and valence with the images presented in the routes. These 48 images were not included in the routes but were used in the recognition task as distractors. The virtual route and the tasks were presented on a big computer screen (32″) in a darkened room by means of the software Open-Sesame.

The combination of images’ valence and type of layout gave rise to three different protocols. Protocol 1 was characterized by layout 1 negative, layout 2 neutral, layout 3 positive; protocol 2 by layout 1 neutral, layout 2 positive, layout 3 negative; protocol 3 by layout 1 positive, layout 2 negative, layout 3 neutral. In order to control possible effects due to the sequence and order of presentation of the movies, six different presentation sequences were created for each protocol. Then, a total of 18 sequences was obtained (3 protocols × 6 sequences). The participants were assigned to the sequences, taking care to have the same number of males and females for each sequence and to have the same number of participants in the three protocols.

#### 2.2.3. Procedure

Participants seated in front of a computer screen and started with filling in a demographic questionnaire (gender and age) and the ten-item personality inventory (TIPI). The total duration of this part ranged between 5 and 10 min. Afterward, participants were requested to perform the Corsi block tapping task (both forward and reversed), the Bergen right-left discrimination task, and a mental rotation task. Finally, participants watched virtual movies one at time. Each participant watched three movies in total—one positive, one negative, and one neutral. At the end of each movie, participants were asked to perform three tasks: a recognition task, a route continuation task, and a comparison task. Once the tasks had been completed, the second movie started. The total duration of the experiment was about 1 h and a half.

##### Questionnaires

Ten-item personality inventory (TIPI): The TIPI is a short personality inventory consisting of ten statements that measure the big five personality traits (i.e., extraversion, agreeableness, conscientiousness, emotional stability, openness). The statements are scored on a 7 point Likert-scale, with 1 as ‘disagree strongly’, and 7 as ‘agree strongly’. One of the statements in the TIPI is: “I see myself as extraverted, enthusiastic”. Every two statements emphasize one personality trait, which is measured by summing up the score and divided by two [30].

##### Cognitive Tasks

**Figure 3:**
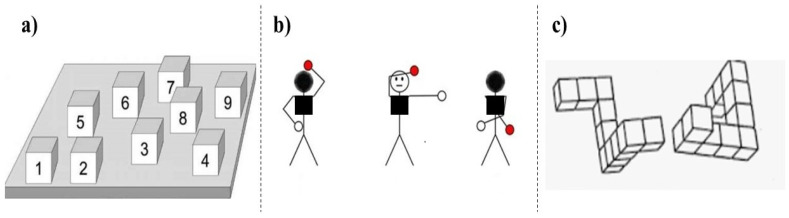
(**a**) The panel of the Corsi block tapping (CBT) is shown from the experimenter’s perspective. The CBT task was performed live by well-trained experimenters. Besides, they had fully memorized all sequences in order to keep timing at a standard pace. The participants did not see the numbers on the blocks; (**b**) An example of the stimuli used in the Bergen right-left discrimination task; (**c**) an example of the stimuli used in the mental rotation task.

Corsi block tapping task: The participants were placed at a table with the Corsi block tapping platform (see Figure 3a). For the first part of the Corsi test, the participants were instructed to mimic the experimenter’s sequence of tapping. The sequence increased as participants correctly reproduced the sequence. In the second part of the Corsi test, the participants were instructed to mimic the experimenter’s sequence of tapping in reversed order. The performance was measured by the product scores (span X points received). 

The Bergen right-left discrimination test: The original paper-pencil version of the Bergen right-left discrimination test [32] was adapted for use on a computer (see Figure 3b). The stimulus set consists of 96 line drawings of a figure with a height of 11 cm. When the head of the figure is highlighted in black, the figure is viewed from the back, so that the left hand of the figure is presented on the left side of the participant. When the head of the figure is highlighted in white, the figure is viewed from the front, so that the left hand of the figure is presented on the right side of the participant. For half of the figures, the left hand is colored red, and for the other half, the right hand is highlighted in red. The participants were asked to state by using a microphone whether the red-circled hand was the right or the left hand. The responses were verbally provided, and the answers were recorded manually by the experimenter. The total of correct answers (mean average, range 0–1) and the response time measured the performance. A total of 68 trials were presented. 

Mental rotation task: The participants were presented with pairs of black figures (1 trial = 2 figures) presented in a canonical orientation or rotated by 90, 135, or 180 degrees. The two figures could be the same, the same but mirrored or different (see Figure 3c). Participants had to indicate whether the two figures were identical or not, as fast and accurate as possible, by using two keys on the keyboard (“1” key: identical; “2” key: not identical). Every trial started with a fixation cross in the middle of the screen (color: black, font: bold, 18; background: white; duration: 1000 ms) after which a pair of block figures was shown in the center of the screen (block figure: black lines; background: white). The pairs of block figures were the same for each participant, while the trials were presented in a random order (total of 52 trials; 4 practice trials). Both response time and accuracy (mean accuracy, range 0–1) were measured. The software E-prime was used to present the stimuli and record the participants’ answers.

##### Landmarks Tasks

Recognition Task: Participants were presented with a total of 16 images—eight that were presented as landmarks in the virtual route, and eight novel images (distractors) one after the other. These novel images had the same semantic content, arousal, and valence of those in the virtual route they viewed. The participants were instructed to use the keyboard to answer and use the button marked with A (‘z’ key) if they did not recognize the image and the button marked with B (‘m’ key) if they did recognize the landmark from the route. The allocation of A/B keys was not counterbalanced. The accuracy was measured. 

**Figure 4:**
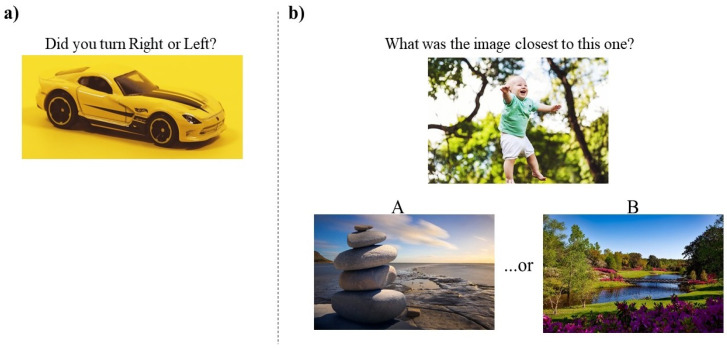
The figure depicts an example of stimuli used as trials in the route continuation task (**a**) and in the distance comparison task (**b**). The images in the figure are not those from the international affective picture system (IAPS), but they are copyright-free images taken from https://www.pexels.com/it-it/. They are present in this figure just for illustration purposes.

Route Continuation Task (RCT): Participants were presented with a landmark from the virtual route and had to answer whether the left or right turn was made when the landmark was encountered (see Figure 4a). The response buttons A (‘z’ key) for a left turn and B (‘m’ key) for a right turn were used to answer. The participants were presented with a total of eight trials. The reaction time and mean accuracy were measured. 

Distance Comparison Task (DCT): Participants were presented with a specific landmark, from the virtual route, and two other used landmarks (A and B) (see Figure 4b). They had to judge what image (A or B) was closest to the target one. The responses were provided by pressing either the A (‘z’ key) or B (‘m’ key) button, corresponding to the landmarks given (A and B). For each target image, three trials with a different level of difficulty were created. The difficulty was based on the distance between the two images, “A and B”. Therefore, in the “difficult” trials, the images A and B were close to each other in the route (e.g., if the target image was the first of the route, then images A and B were, respectively, the seventh and the eighth). In the trials with “medium” difficulties, the images A and B were separated by two or three images (e.g., if the target image was the first of the route, then image A was the fifth and B the eighth). Finally, in the “easy” trials, the images A and B were separated by four or five images (e.g., if the target image was the first of the route, then image A was the third and image B the eighth). Based on this logic, 24 trials were created (8 images × 3 levels of difficulty), and the trials were the same for all the layout x valence combinations; the latter only differed for the kind of images. In other words, the trials’ difficulties were matched across the layouts and were the same for all participants. Each participant performed all the 24 trials. The mean accuracy and reaction time were measured.

### 2.3. Data Analysis

For each variable, the outliers were defined as data values with +/−3.29 standard deviations (cf. [33]). As a consequence, 1% of the data was removed from the entire database. Afterward, the normality assumption for each variable of the study was checked with the z-test ([34]; see Appendix A, Table A2, for the results). In the case of normality assumption violation, the non-parametric tests were used to analyze the data.

First, we verified the presence of differences among the three different conditions (i.e., positive, negative, neutral) for the three landmark tasks (i.e., recognition task, RCT, and DCT), separately. Therefore, two ANOVAs for repeated measures were carried out on the accuracy at the route continuation and distance comparison tasks, respectively. Instead, the Friedman test was used to analyze response times at both tasks and the accuracy at the recognition task. Second and more importantly, we carried out pairwise Pearson correlations (or Spearman correlation in case of not normally distributed data) between TIPI scores, spatial tasks (MR, Corsi, and Bergen R-L), and the three conditions for the route continuation and distance comparison tasks. Since several correlations were carried out, the false discovery rate “Benjamini and Hochberg” (FDR/B–H; [35]) procedure was used to adjust for multiple testing. The adjusted critical *p*-values, henceforth B–H values, were used to assess the statistical significance of each correlation. For the completeness of information, all tests have been shown, but more space has been given to results above the threshold for the comparison for multiple testing.

## 3. Results

### 3.1. Recognition Task

The non-parametric Friedman test on mean accuracy (range 0–1) for the three conditions (positive vs. negative vs. neutral) was carried out. The results showed a high level of accuracy for all the conditions (positive: M = 0.90, SD = 0.10; negative: 0.90, SD = 0.10; neutral: 0.89, SD = 0.11), but no significant difference among the three conditions was observed (chi-square value of 0.96 *p* = 0.62). These results indicated that participants were able to discriminate targets from the distractors in all the conditions highly accurately. This meant that differences among the conditions in the next tasks could not be attributed to the difficulty in remembering the correct images/landmarks. These results have not been discussed further.

### 3.2. Route Continuation Task

#### 3.2.1. Accuracy

The results from ANOVA for repeated measures showed that participants were slightly more accurate in positive than negative and neutral condition (positive: M = 0.75, SD = 0.19; negative: 0.73, SD = 0.19; neutral: 0.72, SD = 0.20), but this difference was not significant: F (2, 258) = 1.14, *p* = 0.32, ƞ^2^_p_ = 0.008.

#### 3.2.2. Response Time

The results from the Friedman test showed no significant differences among conditions (F (2, 258) = 1.17, *p* = 0.31, ƞ^2^_p_ = 0.009).

#### 3.2.3. Correlations

As regards accuracy (see Table 1), the results from the correlation analyses showed significant associations in the positive condition between participants’ performance and mean response time at Bergen R-L test (r = −0.40 *p* < 0.00001; B–H = 0.0007), mean accuracy at Corsi (r = 0.38 *p* < 0.00001; B–H = 0.001) and Corsi reversed (r = 0.33 *p* < 0.00001; B–H = 0.002) tasks, and scores at the agreeableness scale of the TIPI test (r = 0.38 *p* = 0.0001; B–H = 0.003). Furthermore, a significant correlation was observed between response time at the Bergen R-L task and accuracy in neutral condition (r = −0.26 *p* = 0.002; B–H = 0.007). Finally, a significant correlation appeared between scores at the agreeableness scale of the TIPI test and accuracy in the neutral condition (r = 0.24 *p* = 0.005; B–H = 0.009).

As regards response time (see Table 2), no significant correlations appeared. However, it was interesting to notice a positive association between response time in the negative condition and scores at extraversion (r = 0.16 *p* = 0.06; B–H = 0.008).

### 3.3. Distance Comparison Task

#### 3.3.1. Accuracy

The results from ANOVA for repeated measures showed a significant difference among the three conditions. The Tukey post hoc test indicated that participants were more accurate in positive than negative and neutral condition (at least *p* < 0.05) (positive: M = 0.68, SD = 0.16; negative: 0.62, SD = 0.15; neutral: 0.61, SD = 0.13) (F(2, 270) = 11.37, *p* = 0.00002, ƞ^2^_p_ = 0.07).

#### 3.3.2. Response Time

The results from the Friedman test showed no significant differences among conditions (F < 1).

#### 3.3.3. Correlations

As regards accuracy (see Table 3), significant correlations were found between response time at mental rotation task and performance in the positive (r = −0.35 *p* = 0.001; B–H = 0.002) condition and between accuracy at mental rotation task and performance in the neutral condition (r = 0.27 *p* = 0.001; B–H = 0.002).

As regards response times (see Table 4), the results showed significant associations between response time at mental rotation task and response time in negative (r = 0.34 *p* < 0.00001; B–H = 0.0006), neutral (r = 0.35 *p* < 0.00001; B–H = 0.001), and positive (r = 0.39 *p* < 0.00001; B–H = 0.002) conditions. Although not statistically significant, it was interesting to notice a positive association between response time in the negative condition and scores at emotional stability (r = 0.20 *p* = 0.02; B–H = 0.006) and openness (r = 0.19 *p* = 0.03; B–H = 0.007).

In brief, the analyses revealed that:

(i) The participants were better at judging distances among landmarks in positive as compared to the negative and neutral conditions. No effect of emotions was found in the route continuation task;
(ii) As regards the route continuation task, the role of basic spatial abilities and personality traits specifically emerged in the positive, and to a less extent, in the neutral condition: the faster and more accurate participants were at the B-RL test, the more accurate they were in judging if at specific landmarks they did right or left. Similarly, the more they declared to be friendly/compassionate, the more accurate they were in the route continuation task. Furthermore, specific positive associations appeared between performance at the Corsi test and performance in the positive condition: the higher the performance at the Corsi test (both normal and reversed), the more accurate the performance at route continuation task;
(iii) As regards the distance comparison task, the role of spatial abilities emerged for all the three conditions, but again to a greater extent in the positive and neutral condition as compared to the negative one. Overall, the faster and more accurate participants were in the mental rotation task, the faster they were in judging correctly the distances among landmarks. Instead, the correlations with a tendency towards significance appeared between emotional stability, openness, and performance in the negative condition. Specifically, the higher the emotional stability and openness to experience, the greater the time needed to evaluate distances between negative stimuli.

## 4. Discussion

This research work was the first to explore the possible role of individual factors, both in terms of basic spatial abilities and personality traits, in the spatial representation of emotionally laden landmarks. To this aim, we asked participants to watch movies of virtual walks along routes characterized by positive, negative, or neutral landmarks. Afterward, they were asked to indicate if, at a specific landmark, they did left or right (route continuation task) and to judge the relative distances between landmarks (distance comparison task). Besides, the individual factors were assessed by asking participants to complete a questionnaire to measure personality traits (TIPI) and to perform three cognitive tasks: a mental rotation task (MR), a task measuring the sequential memory of positions (i.e., Corsi and Corsi reversed), and a task that evaluated the ability to discriminate right from left (i.e., B-RL).

As regards the relationships between individual factors and spatial representations of emotionally laden landmarks, overall results showed significant relationships in the positive and, to a less extent, in the neutral condition. Specifically, if the route was characterized by positive landmarks, then as the ability to correctly remember sequences of positions (i.e., highest score at Corsi and Corsi reversed) increased, so did the accuracy at the route continuation task. This result was in line with several studies that indicate a positive correlation between performance at Corsi task and navigational skills [21,36,37,38,39]. Similarly, the results showed that the faster the right-left discrimination task was performed, the more accurate the performance at the route continuation task was in both positive and neutral conditions. This was a new and interesting result because, for the first time, a positive relationship between left/right discrimination and navigational skills was shown. According to Hjelmervik and colleagues ([22]; see also [40]), the reason for this effect could be that the ability to discriminate right from left stems from environmental navigation. In fact, the right and left positions vary according to the position that our body assumes in space. Another possibility is that this association is due to the fact that the correct execution of the Bergen R-L test requires a fundamental skill for effective navigation in the environment, namely mental rotation [9,10,11,12]. However, this hypothesis is not supported by our results, where an association between mental rotation and performance in the route discrimination tasks did not appear. Instead, a strong association between performance at mental rotation task and the distance comparison task appeared. In this case, the faster the participants were in performing the mental rotation task correctly, the faster they were in judging/comparing distances between landmarks. This association appeared for the three emotional conditions, but again to a wider extent in the positive and neutral conditions.

The question at this point is: why has the association between basic spatial abilities and cognitive tasks appeared more in the positive than in the neutral condition and even less in the negative one? It is possible to think that the different cognitive processes were used in the positive, neutral, and negative conditions to perform both route continuation and distance comparison tasks. Specifically, the positive condition seemed to have favored the use of strategies based on visual-spatial working memory, such as mental rotation and visuospatial working memory. This was supported by several studies, showing that a mildly positive mood improves performance in working memory (e.g., [19,41,42]; for reviews: [43,44]) and the performance in tasks that call for creative solutions, innovative problem solving, and coping skills [45,46,47,48,49,50,51,52]. The reason for this can be found in the “neuropsychological theory of positive mood” [53]. According to Ashby and colleagues [53], the positive mood increases dopamine, which is an important underlying biological mechanism for executive control and working memory (WM).

Another interesting result obtained with this study is represented by the relationships between personality traits and spatial memory tasks. Specifically, as the scores on the agreeableness scale, which is a tendency to be altruistic, trusting, modest, and compliant [54,55], increased, so did the accuracy at the route continuation task in the positive condition and, to a less extent, in the neutral condition. These results seemed to be also in line with what found by Pazzaglia and colleagues [24]. However, literature provides no conceptual rationale for a relationship between agreeableness and cognitive ability in general (e.g., [56,57,58]). Since this association appeared to be particularly significant in the positive and neutral condition but not in the negative one, it is possible that individuals with higher levels of agreeableness could be less prone to process and remember negative stimuli [59]. In line with this, it has been shown that people who are higher in agreeableness show higher emotional sensitivity, lower variability of sadness, and more positive subjective evaluations of daily incidents [27]. In contrast, a positive relationship appeared between emotional stability and openness and response times at the distance comparison task in the negative condition: The higher scores at emotional stability (i.e., low neuroticism) and openness scales, the more time to perform the distance comparison task. This result was probably due to the fact that less neurotic people tend to be less influenced by negative stimuli than more neurotic people [60]. This, in turn, could have led to less emphasis on the emotional content of negative landmarks. However, these latter results should be taken carefully as they did not survive correction for multiple comparisons.

Finally, as regards the influence of emotions on spatial representations, the results showed that the emotional valence of the landmarks did not influence the participants’ performance in the route continuation but only in the distance comparison task. Specifically, participants were more accurate in judging what landmark was closest to the target one, especially when dealing with positive rather than neutral and negative landmarks. These results supported our hypotheses for the distance comparison task but not for the route continuation task. In fact, we expected participants to memorize more accurately the landmarks’ directional information, especially when the latter was negative rather than neutral or positive. This is because, as stated also by Chan and colleagues [61], memorization of spatial information of negative landmarks should have an evolutionary/adaptive relevance, i.e., it is safer to remember spatial information about negative elements in order to avoid them in future explorations. Instead, in contrast with previous evidence (e.g., [2,61]), we did not find negative images improving memorization of spatial information. We argued that this was due to the fact that the negative images we used were not as impressive or scary as compared to those used in other studies. In fact, Chan and colleagues [61] used pictures of “mutilated bodies” and explicit “scenes of violence” as negative images. These negative stimuli could, in turn, have had a stronger impact on participants’ spatial memory, and, contrary to what we found, could have revealed major differences among negative, positive, and neutral conditions.

On the other side, the advantage we observed for the positive condition in the distance comparison task was in line with the evidence shown in past studies but also contained elements of novelty. In fact, several studies have shown that a positive mood has beneficial effects on visual-spatial tasks (e.g., [3,4]). For example, Ruotolo and colleagues [5] showed that participants were more accurate in imagining the distances between different landmarks when they had a positive rather than negative or neutral value. However, the task used by Ruotolo and colleagues [5] required a more egocentric or body-based strategy to be performed, as participants were asked to imagine themselves walking from one landmark to another. Instead, here for the first time, we showed that the presence of positive landmarks facilitated the use of a more allocentric strategy. In fact, in the distance comparison task, the three landmarks were shown to the participant as if they were a sort of abstract map, and their task was to compare the distances between these landmarks regardless of the participants’ position.

Before concluding, it is important to indicate some of the limitations of the current study. First, some relevant factors or individual differences that might also play a fundamental role in the way we represented spatial information were not considered. For example, several studies have shown differences related to gender (e.g., [62,63,64,65,66,67]) in combination with age (e.g., [68,69]), spatial experience, and familiarity with the environment [67,70,71,72,73,74]. Second, this study shows some of the most important factors associated with processing of positive stimuli, but unfortunately failed to shed light on what strategies might be used in the condition with negative stimuli. Third, the correlations were limited to the two spatial judgment tasks used in this study. Therefore, future studies should assess the weight of other individual factors by using also other kinds of spatial tasks that assess other aspects of route representations.

## 5. Conclusions

Overall, the results of this study indicated that emotions have a far greater influence on the way humans represent space information than is actually recognized. They can influence the cognitive mechanisms that underpin the processing of spatial information. Furthermore, individuals may be more or less sensitive to the emotional content of landmarks, depending on their personality. Therefore, future studies about emotions and spatial cognition must take into account individual differences, both in terms of different levels of cognitive abilities and participants’ personality profiles. 

## Figures and Tables

**Table 1:** The table shows the correlation indexes (and the corresponding *p*-levels) between cognitive tasks, personality traits, and accuracy at the route continuation task. Pearson correlations (r·P) are reported in bold. The Spearman correlations (R·S) are not in bold. The asterisk (*) indicates the correlations that survived to the correction for multiple comparisons.

	Route Continuation Task- Accuracy					
	Negative		Neutral		Positive	
	r·P/ R·S	*p*-level	r·P/ R·S	p-Level	r·P/ R·S	*p*-Level
B-RL_ACC	0.06	0.504	0.13	0.13	0.14	0.12
B-RL_RT	−0.15	0.078	−0.26 *	0	−0.40 *	0
**CBT**	**0.16**	**0.057**	**0.09**	**0.28**	**0.38** *	**0**
**CBT_REV**	**0.15**	**0.079**	**0.18**	**0.04**	**0.33** *	**0**
**MR_ACC**	**0.12**	**0.174**	**0.06**	**0.50**	**−0.04**	**0.64**
MR_RT	−0.17	0.055	−0.13	0.13	−0.05	0.60
**TIPI_AG**	**0.20**	**0.022**	0.24 *	**0**	**0.38** *	**0**
TIPI_ES	−0.04	0.643	−0.04	0.63	−0.01	0.89
**TIPI_EX**	**−0.04**	**0.680**	**−0.11**	**0.22**	**0.01**	**0.89**
TIPI_OP	−0.02	0.790	−0.04	0.68	−0.08	**0.39**
**TIPI_CO**	**0**	**0.963**	**−0.02**	**0.79**	**0**	**0.96**

Abbreviations: ACC = accuracy; RT = response time; B-RL = Bergen right/left test; CBT = Corsi block test; REV = reversed; MR = mental rotation; TIPI= ten item personality inventory; AG = agreeableness; CO = conscientiousness; ES = emotional stability; EX = extraversion; OP = openness.

**Table 2:** The table shows Spearman’s (R) correlation indexes (and the corresponding *p*-levels) between cognitive tasks, personality traits, and accuracy at the route continuation task.

	Route Continuation Task- Response Time					
	Negative		Neutral		Positive	
	R·S	*p*-Level	R·S	*p*-Level	R·S	*p*-Level
B-RL_ACC	0.058	0.510	0.093	0.293	−0.086	0.327
B-RL_RT	0.145	0.097	0.086	0.332	0.088	0.311
CBT	−0.119	0.168	0.022	0.799	0.062	0.476
CBT_REV	−0.083	0.337	−0.011	0.896	−0.026	0.763
MR_ACC	0.020	0.816	0.153	0.078	−0.105	0.228
MR_RT	0.156	0.071	−0.006	0.947	0.120	0.168
TIPI_AG	−0.045	0.606	−0.024	0.784	−0.044	0.615
TIPI_CO	0.092	0.287	0.148	0.088	0.046	0.595
TIPI_ES	0.047	0.584	0.047	0.586	−0.030	0.728
TIPI_EX	0.160	0.063	−0.112	0.198	−0.051	0.552
TIPI_OP	0.021	0.805	0.048	0.585	−0.036	0.674

Abbreviations: ACC = accuracy; RT = response time; B-RL = Bergen right/left test; CBT = Corsi block test; REV = reversed; MR = mental rotation; AG = agreeableness; CO = conscientiousness; ES = emotional stability; EX = extraversion; OP = openness.

**Table 3:** The table shows the correlation indexes (and the corresponding *p*-levels) between cognitive tasks, personality traits, and accuracy at the distance comparison task. Pearson correlations (r·P) are reported in bold. The Spearman correlations (R·S) are not in bold. The asterisk (*) indicates the correlations that survived to the correction for multiple comparisons.

	Distance Comparison Task- Accuracy					
	Negative		Neutral		Positive	
	r·P/R·S	*p*-Level	r·P/R·S	*p*-Level	r·P/R·S	*p*-Level
B-RL_ACC	0.15	0.09	0.20	0.02	−0.02	0.79
B-RL_RT	−0.21	0.01	−0.01	0.94	−0.01	0.91
**CBT**	**0.13**	**0.12**	**0.11**	**0.20**	**−0.10**	**0.23**
**CBT_REV**	**0.18**	**0.04**	**0.16**	**0.07**	**−0.06**	**0.51**
**MR_ACC**	**0.12**	**0.17**	0.27 *	**0**	**0.09**	**0.30**
MR_RT	−0.18	0.03	−0.08	0.36	−0.35 *	0
**TIPI_AG**	**0.12**	**0.15**	**0.09**	**0.32**	**0.06**	**0.48**
TIPI_ES	0.11	0.22	0.13	0.12	0	0.96
**TIPI_EX**	**−0.11**	**0.21**	**−0.10**	**0.23**	**−0.06**	**0.48**
TIPI_OP	0.13	0.14	0.15	0.08	**−0.06**	**0.51**
**TIPI_CO**	**−0.06**	**0.51**	**−0.12**	**0.16**	0.02	0.85

Abbreviations: ACC = accuracy; RT = response time; B-RL = Bergen right/left test; CBT = Corsi block test; REV = reversed; MR = mental rotation; TIPI= ten item personality inventory; AG = agreeableness; CO = conscientiousness; ES = emotional stability; EX = extraversion; OP = openness.

**Table 4:** The table shows Spearman’s (R) correlation indexes (and the corresponding *p*-levels) between cognitive tasks, personality traits, and accuracy at the distance comparison task. The asterisk (*) indicates the correlations that survived to the correction for multiple comparisons.

	Distance Comparison Task- Response Time					
	Negative		Neutral		Positive	
	R·S	*p*-Level	R·S	*p*-Level	R·S	*p*-Level
B-RL_ACC	0	1.00	−0.02	0.80	0.01	0.93
B-RL_RT	−0.01	0.90	0.01	0.87	−0.06	0.46
CBT	−0.07	0.43	−0.01	0.86	0.06	0.49
CBT_REV	−0.02	0.85	0.02	0.82	0.01	0.95
MR_ACC	−0.12	0.17	−0.03	0.76	−0.13	0.14
MR_RT	0.34 *	0	0.35 *	0	0.39 *	0
TIPI_AG	0.01	0.95	−0.14	0.10	−0.03	0.76
TIPI_CO	−0.06	0.49	0.07	0.43	−0.04	0.62
TIPI_ES	0.20	0.02	0.13	0.14	0.06	0.50
TIPI_EX	0.08	0.38	−0.09	0.28	0.05	0.53
TIPI_OP	0.19	0.03	0.11	0.20	0.05	0.60

Abbreviations: ACC = accuracy; RT = response time; B-RL = Bergen right/left test; CBT = Corsi block test; REV = reversed; MR = mental rotation; TIPI = Aten item personality inventory; AG = agreeableness; CO = conscientiousness; ES = emotional stability; EX = extraversion; OP = openness.

## Appendix A

**Table A1:** Valence and arousal values of the images put along the routes (the code is taken by the IAPS).

			Mean	
	*Landmark*	*Code*	*Valence*	*Arousal*
**Negative**	AimedGun	6230	2.37	7.35
	Attack	6550	2.73	7.09
	Attack	6510	2.46	6.96
	KKKrally	9810	2.09	6.62
	Gang	6821	2.38	6.29
	Fire	9623	3.04	6.05
	Skinhead	9800	2.04	6.05
	Snake	1022	4.26	6.02
	Terrorist	6213	2.91	5.86
	Dog	1301	3.7	5.77
	Bomb	2692	3.36	5.35
	Toddler	2095	1.79	5.25
**Positive**	Roller	8492	7.21	7.31
	Lightning	5950	5.99	6.79
	Sailing	8080	7.73	6.65
	Water Skier	8200	7.54	6.35
	EroticCouple	4608	7.07	6.47
	Money	8501	7.91	6.44
	Romantic	4597	6.95	5.91
	Wedding	4626	7.6	5.78
	Fireworks	5480	7.53	5.48
	Baby	2045	7.87	5.47
	Pregnant	2155	6.78	5.43
	Puppies	1710	8.34	5.41
**Neutral**	Lamp	7175	4.87	1.72
	Chess	2580	5.71	2.79
	Couple	2396	4.91	3.34
	Boy	2280	4.22	3.77
	Hammer	7110	4.55	2.27
	Tissue	7950	4.94	2.28
	Window	7490	5.52	2.42
	Baskets	7041	4.99	2.6
	Basket	7010	4.94	1.76
	Fan	7020	4.97	2.17
	Fork	7080	5.27	2.32
	RollingPin	7000	5.00	2.42
	NeutGirl	2440	4.49	2.63
	Girl	2411	5.07	2.86
	Flowers	5731	5.39	2.74
	Bridge	7547	5.21	3.18
	Spoon	7004	5.04	2.00
	Shoes	7031	4.52	2.03
	Bowl	7006	4.88	2.33
	Building	7491	4.82	2.39
	Man	2190	4.83	2.41
	Golf	8312	5.37	3.32
	NeuWoman	2038	5.09	2.94
	Farmer	2191	5.3	3.61

**Table A2:** The table reports the skewness and kurtosis values for all the variables of the study. A *z*-test was applied for normality test using the skewness and kurtosis values. The *z*-score was obtained by dividing the skew values and kurtosis by their standard errors (see third and sixth columns). In line with West et al.’s suggestions (1996), all the absolute *z*-scores over 3.29, which corresponds with an alpha level of 0.05, would suggest a non-normal distribution. Therefore, we indicated with one asterisk (*) all variables whose distribution did not substantially differ from normality, with (**) all distributions that slightly differed from normality, with (***) all distributions with severe departure from normality. Abbreviations: Acc = accuracy; RT = response time; B-RL = Bergen right/left test; CBT = Corsi block test; REV = reversed; TIPI = Ten item personality inventory; AG = agreeableness; CO = conscientiousness; ES = emotional stability; EX = extraversion; OP = openness; Neg = negative; Neu = neutral; Pos = positive; Cont = route continuation task; Comp = distances comparison task.

	*Skewness (S)*	*Std. Err.*	*S/Std. Err.*	*Kurtosis (K)*	*Std. Err.*	*K/Std. Err.*
*B-RL_Acc ****	−2.045	0.209	9.806	3.650	0.414	8.814
*B-RL_RT ***	1.034	0.209	4.959	0.198	0.414	0.479
*CBT **	0.386	0.206	1.869	−0.200	0.410	0.488
*CBT_REV **	0.073	0.206	0.355	−0.323	0.410	0.788
*MR_Acc **	−0.134	0.208	0.646	−0.833	0.413	2.019
*MR_RT ***	0.767	0.208	3.694	0.475	0.413	1.150
*TIPI_AG **	−0.240	0.206	1.165	0.459	0.410	1.121
*TIPI_CO **	−0.471	0.206	2.285	−0.349	0.410	0.852
*TIPI_ES ***	−0.732	0.206	3.547	−0.096	0.410	0.235
*TIPI_EX **	0.043	0.206	0.208	−0.981	0.410	2.395
*TIPI_OP ***	−0.682	0.206	3.305	−0.348	0.410	0.849
*Neg_Rec_Acc ****	−1.067	0.207	5.152	0.837	0.411	2.036
*Neu_Rec_Acc ***	−0.975	0.208	4.693	0.321	0.413	0.777
*Pos_Rec_Acc ****	−1.108	0.210	5.274	0.755	0.417	1.811
*Neg_Comp_Acc **	0.108	0.208	0.518	−0.204	0.413	0.494
*Neg_Comp_RT ***	1.025	0.208	4.935	1.112	0.413	2.694
*Neg_Cont_Acc **	−0.372	0.208	1.789	−0.877	0.413	2.126
*Neg_Cont_RT ****	1.232	0.208	5.930	1.440	0.413	3.490
*Neu_Comp_Acc **	0.069	0.207	0.335	−0.302	0.411	0.733
*Neu_Comp_RT ****	1.293	0.207	6.245	1.416	0.411	3.444
*Neu_Cont_Acc **	−0.306	0.209	1.463	−0.967	0.416	2.327
*Neu_Cont_RT ****	1.659	0.209	7.928	3.045	0.416	7.326
*Pos_Comp_Acc **	0.095	0.206	0.461	−0.702	0.410	1.713
*Pos_Comp_RT ***	0.803	0.206	3.893	0.051	0.410	0.125
*Pos_Cont_Acc **	−0.551	0.208	2.652	−0.261	0.413	0.633
*Pos_Cont_RT ****	1.770	0.208	8.520	3.273	0.413	7.932
